# Roles of Toll-like Receptor Signaling in Inflammatory Bone Resorption

**DOI:** 10.3390/biology13090692

**Published:** 2024-09-04

**Authors:** Tsukasa Tominari, Chiho Matsumoto, Yuki Tanaka, Kensuke Shimizu, Masaru Takatoya, Moe Sugasaki, Kento Karouji, Urara Kasuga, Chisato Miyaura, Shinji Miyata, Yoshifumi Itoh, Michiko Hirata, Masaki Inada

**Affiliations:** 1 Department of Biotechnology and Life Science, Tokyo University of Agriculture and Technology, 2-24-16 Nakacho, Koganei-shi, Tokyo 184-8588, Japan; tominari@ncnp.go.jp (T.T.); c-matsu@cc.tuat.ac.jp (C.M.); miyaura@isc.chubu.ac.jp (C.M.); hirata@cc.tuat.ac.jp (M.H.); 2 Cooperative Major of Advanced Health Science, Tokyo University of Agriculture and Technology, 2-24-16 Nakacho, Koganei-shi, Tokyo 184-8588, Japanflowervv.pku1018@gmail.com (K.K.); 3 Inada Research Unit, Institute of Global Innovation Research, Tokyo University of Agriculture and Technology, 2-24-16 Nakacho, Koganei-shi, Tokyo 184-8588, Japan; smiyata@go.tuat.ac.jp (S.M.); yoshi.itoh@kennedy.ox.ac.uk (Y.I.); 4 Kennedy Institute of Rheumatology, Nuffield Department of Orthopaedics, Rheumatology and Musculoskeletal Sciences, University of Oxford, Oxford OX3 7FY, UK

**Keywords:** Toll-like receptors, innate immunity, bone resorption, osteoblasts, osteoclasts, prostaglandin E2, periodontal disease

## Abstract

**Simple Summary:**

Toll-like receptor (TLR) signaling contributes to the pathogenesis of inflammatory oral diseases, such as periodontal disease by stimulating osteoclast differentiation and function. Recent reports suggest that various components from bacteria, viruses, and autologous cells act as TLR ligands. Lipopolysaccharide (LPS), a TLR4 ligand, induces osteoclast differentiation and bone resorption; however, blocking PGE2 synthesis or antagonizing PGE2 receptor signaling abrogates LPS-induced inflammatory bone resorption. In addition, other ligands for TLR2/1, TLR2/6 and TLR3 facilitate PGE2 production, leading to osteoclastic bone resorption. This review introduces the latest findings regarding the relationship between TLR signaling pathways and inflammatory bone resorption.

**Abstract:**

Toll-like receptors (TLRs) are pattern recognition receptors expressed in immune cells, including neutrophils, macrophages, and dendritic cells. Microbe-associated molecular patterns, including bacterial components, membranes, nucleic acids, and flagella are recognized by TLRs in inflammatory immune responses. Periodontal disease is an inflammatory disease known to cause local infections associated with gingival inflammation, subsequently leading to alveolar bone resorption. Prostaglandin E2 (PGE2) is a key mediator of TLR-induced inflammatory bone resorption. We previously reported that membrane-bound PGE synthase (mPGES-1)-deficient mice failed to induce bone resorption by lipopolysaccharide (LPS), a major pathogenic factor involved in periodontal bone resorption. Further experiments exploring specific pathogen-promoting osteoclast differentiation revealed that various TLR ligands induced osteoclast differentiation in a co-culture model. The ligands for TLR2/1, TLR2/6, TLR3, and TLR5, as well as TLR4, induce osteoclast differentiation associated with the production of PGE2 and the receptor activator of nuclear factor-kappa B ligand (RANKL), an inevitable inducer of osteoclast differentiation in osteoblasts. In vivo, local injection of TLR ligands, including TLR2/1, TLR2/6, and TLR3, resulted in severe alveolar bone resorption. This review summarizes the latest findings on TLR-mediated osteoclast differentiation and bone resorption in inflammatory diseases, such as periodontal diseases.

## 1. Introduction

Toll-like receptors (TLRs) are pattern recognition receptors (PRRs) that recognize microbe-associated molecular patterns (MAMPs), also known as pathogen-associated molecular patterns (PAMPs), and damage-associated molecular patterns (DAMPs) that initiate innate responses in immune cells, including neutrophils, macrophages, and dendritic cells [1].

**Figure 1:**
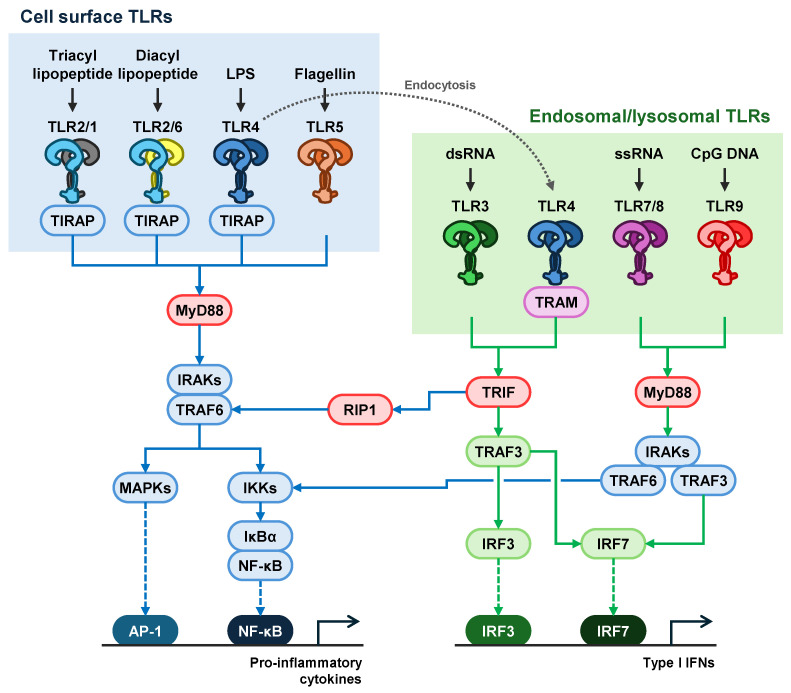
TLR signaling pathways. TLR2/1, TLR2/6, and TLR4 transduce the TIRAP/MyD88-dependent pathway to activate NF-κB and AP-1 transcription, which induces the production of pro-inflammatory cytokines. TLR5, TLR7/8, and TLR9 activate the MyD88-dependent pathway without the TIRAP adaptor protein. In contrast, TLR3 and endocytosed TLR4 activate a TRIF-dependent pathway to activate both NF-κB and IRFs. Endocytosed TLR4 requires a TRAM adaptor protein. TLR7/8 and TLR9 also transduce the IRF7 and NF-κB pathways.

Thus far, 10 TLRs have been identified in humans (TLR1-10) and 12 TLRs in mice (TLR1-9 and 11-13) [2]. The TLR family can be divided into cell surface and intracellular. TLR1, TLR2, TLR4, TLR5, and TLR6 are mainly located on the cell surface of the plasma membranes, whereas TLR3, TLR7, TLR8, and TLR9 are primarily located on the intracellular membranes of endosomes, phagosomes, and lysosomes. All TLRs except TLR3 activate the myeloid differentiation factor 88 (MyD88)-dependent pathway. In contrast, TLR3 and TLR4 activate toll/interleukin-1 receptor-domain-containing adapter-inducing interferon-β (TRIF)-dependent pathways. Because TLR4 is internalized into the cytosol and located on intracellular membranes, it transduced both MyD88-dependent and TRIF-dependent signaling pathways. These pathways stimulate nuclear factor-κB (NF-κB), mitogen-activated protein kinases (MAPKs), and interferon regulatory factors (IRFs) to produce inflammatory cytokines and type I interferons (IFNs) (Figure 1). TLRs mediate several cases of oral infection, such as hard tissue breakdown of alveolar bones in periodontal disease.

Bone metabolism is precisely regulated by the balance between osteoclastic bone resorption and osteoblastic bone formation, known as bone remodeling [3]. Various factors, including inflammatory cytokines, hormones, mechanical unloading, and aging, induce excessive osteoclastic bone resorption, resulting in decreased bone mineral density (BMD) and bone volume [4,5]. Osteoclasts are multinucleated bone-resorbing cells that differentiate from macrophage-lineage cells. In contrast, osteoblasts play a dual role in forming new bone through the secretion of bone substrates and the induction of osteoclast differentiation through the expression of receptor activator of NF-κB ligand (RANKL). The interaction between RANKL and RANK expressed on osteoclast precursor cells stimulates osteoclast differentiation by activating several pathways, such as the nuclear factor of activated T cells (NFATc1), NF-κB, and activator protein (AP)-1 [6].

Periodontal disease is an inflammatory disease characterized by a bacterial infection that results in the progression of alveolar bone resorption and tooth loss. Lipopolysaccharide (LPS), an outer membrane component of Gram-negative bacteria, is a well-characterized TLR4 ligand that induces inflammatory bone resorption in periodontal diseases. Various bacterial and virus components and cellular components from dead cells are identified as exogenous and endogenous TLR ligands, respectively [7,8,9]. Because these ligands are considered to be abundant in infiltrated tissues, TLR signaling can be excessively activated and induce inflammatory bone resorption.

In this review, we summarize the role of TLRs in inflammatory bone resorption. 

## 2. Prostaglandin E2 Is a Primary Mediator of Inflammatory Bone Resorption

PGE2 is a primary inflammatory mediator of lipid metabolites that is synthesized via the arachidonic acid (AA) cascade and is a potent inducer of osteoclastic bone resorption in inflammatory bone-related diseases, such as periodontitis and arthritis [10,11,12]. The AA cascade proceeds through metabolic processes: cell membrane phospholipids release AA via phospholipase A2 (PLA2). AA is converted to PGG2 and PGH2 by cyclooxygenases (COXs), and PGE synthases produce PGE2 from PGH2. PGE2 receptors are classified into four G-protein-coupled receptor subtypes, EP1-4. PGE2 synthesis (production) is stimulated by several cytokines, such as interleukin-1 (IL-1) and tumor necrosis factor-α (TNF-α), through the upregulation of cyclooxygenase 2 (COX2) and membrane-bound PGE synthase-1 (mPGES-1) [13].

We herein report a role for PGE2 in inflammatory bone resorption. Adding PGE2 or each agonist to EP2 and EP4, but not EP1 and EP3, induced osteoclast differentiation in mouse bone marrow cultures and bone resorption in mouse calvarial organ cultures [14]. In addition, PGE2-induced bone resorption was attenuated only in EP4 knockout mice, not in EP1-3 knockout mice, in mouse calvarial organ cultures [15]. Other studies have consistently reported that PGE2 enhances RANKL-induced osteoclast differentiation [16,17]. In fact, in an animal model of periodontitis, the blockage of PG biosynthesis by indomethacin suppressed alveolar bone resorption [18,19]. We also clarified the crosstalk between TLR signaling and PGE2 in osteoclast differentiation [11,20,21,22].

## 3. Roles of TLR Signaling in Osteoclast Differentiation

### 3.1. Exogenous and Endogenous Ligands for TLRs

Various MAMPs and DAMPs are identified as TLR ligands (Table 1). TLRs form hetero- or homodimers to activate downstream signaling pathways (Figure 1). TLR2/1, TLR2/6, and TLR4 recruit cytoplasmic TIR-domain-containing adaptor protein (TIRAP) and activate the MyD88-dependent pathway. TLR5, TLR7, TLR8, and TLR9 use the MyD88-dependent pathway, whereas TLR3 uses a TRIF-dependent pathway. In particular, TLR4 is endocytosed and translocated on an endosomal membrane, similar to TLR3, TLR7, TLR8, and TLR9, to activate the TRIF-related adaptor molecule (TRAM) and TRIF-dependent pathways. The MyD88-dependent pathway drives the induction of proinflammatory cytokines via NF-κB and AP-1, whereas the TRIF-dependent pathway drives type 1 IFNs via IRF3 and IRF7. Proinflammatory cytokines such as IL-1 and tumor necrosis factor-α (TNF-α) are positive regulators of osteoclast differentiation and function by acting on osteoblasts, osteoclast precursor cells, and mature osteoclasts [23,24]. Type 1 interferons, such as IFN-α and IFN-β, are negative regulators of osteoclast differentiation and function [25].

### 3.2. Roles of Cell Surface and Intracellular TLR4 for LPS from Gram-Negative Bacteria

**Figure 2:**
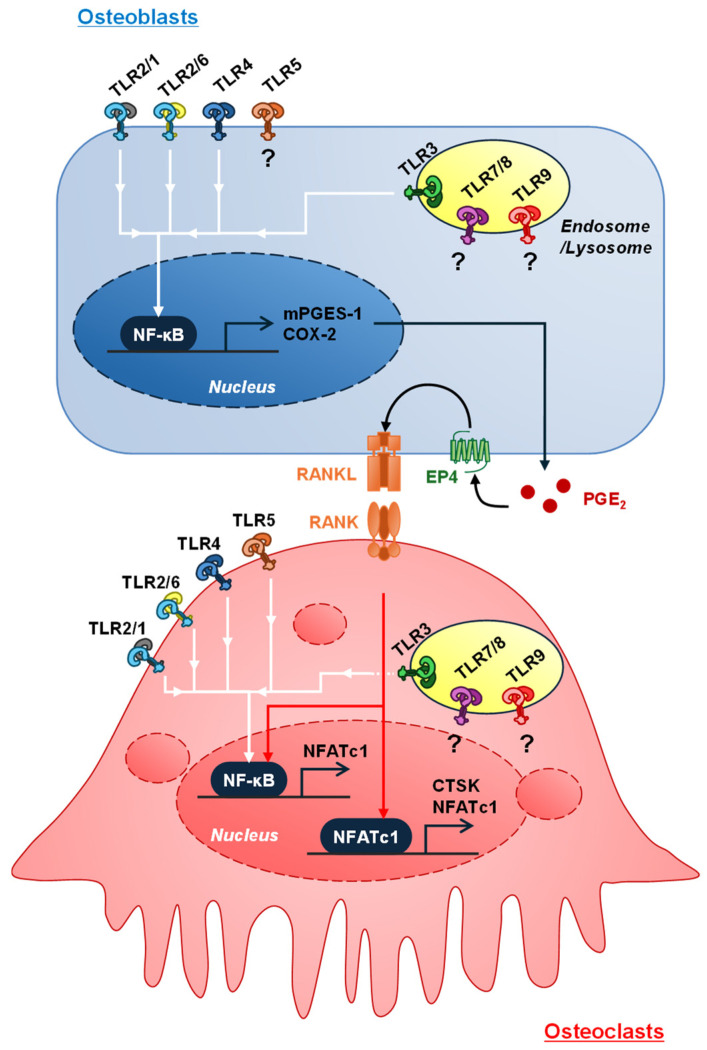
Roles of TLR signaling in osteoclast differentiation. In osteoblasts, TLR2/1, TLR2/6, TLR3, and TLR4 signaling activates the NF-κB pathway, leading to PGE2 production mediated by COX2 and mPGES-1. PGE2 activates EP4 signaling in an autocrine/paracrine manner, followed by RANKL expression. In addition, these signaling pathways directly activate the osteoclast function and survival via the NF-κB pathway. Both indirect and direct effects of TLR signaling induce osteoclastic bone resorption.

LPS is a bacterial endotoxin that is an outer membrane component of Gram-negative bacteria and is a major pathogenic factor in periodontal disease. TLR4 is a well-characterized LPS receptor. Gram-negative bacteria are found in subgingival plaques deep in the periodontal pocket. Periodontal pathogens such as *Aggregatibacter actinomycetemcomitans* and *P. gingivalis* possess LPS and cause severe inflammatory responses when they infect periodontal tissues. TLR4 requires an accessory protein, myeloid differentiation protein 2 (MD2), to recognize LPS. LPS-binding protein (LBP) forms a complex with LPS, binds to CD14, and is subsequently delivered to the TLR4-MD2 complex to activate TLR4 signaling [26]. Cell surface TLR4-MyD88-dependent signaling leads to early NF-κB activation. At this point, TLR4 is subsequently endocytosed, which is controlled by CD14, and intracellular (endosomal) TLR4 transduces TRIF-dependent signaling, resulting in late NF-κB activation [27]. We previously demonstrated that LPS upregulated the mRNA expression of *Ptgs2* (COX-2) and *Ptges* (mPGES-1) and promoted PGE2 synthesis in osteoblasts, inducing osteoclast differentiation through PGE2-mediated *Tnfsf11* (RANKL) upregulation in osteoblasts [28]. Intraperitoneal injection of LPS into cytosolic PLA2α (cPLA2α)-deficient mice resulted in decreased PGE2 levels in BMCs but failed to decrease femoral BMD [28]. Similarly, local injection of LPS into mouse mandibular gingiva induced alveolar bone resorption characterized by a decrease in alveolar BMD, whereas LPS injection did not decrease alveolar BMD in mPGES-1-deficient mice [11]. We further confirmed that blocking PGE2 production using several compounds and natural phytochemicals, including β-cryptoxanthin [29,30], lutein [31], epigallocatechin gallates [32,33], and polymethoxyflavones [34,35], significantly suppressed LPS-induced osteoclast differentiation and alveolar bone resorption. Based on these data, we suggest that LPS targets osteoblasts expressing TLR4 and stimulates PGE2 production by upregulating *Ptgs2* and *Ptges* mRNA expression via the LPS/TLR4/NF-κB pathway. PGE2 binds to EP4 via paracrine and autocrine signaling in osteoblasts to induce RANKL expression, supporting osteoclast differentiation (Figure 2).

LPS also directly controls the functions of osteoclast precursor cells and mature osteoclasts. Several reports have shown that LPS plays a dual role in osteoclast differentiation, inducing osteoclast differentiation without RANKL [36,37], promoting osteoclast differentiation via the TLR4/TNF-α axis in RANKL-primed cells [38,39], and prolonging the lifespan of mature osteoclasts [40,41]. In contrast, LPS suppresses RANKL-induced osteoclast differentiation [39,40]. Itoh et al. [41] reported that the LPS-mediated survival of osteoclasts does not mediate the production of cytokines, including IL-1 and TNF-α, osteoprotegerin (OPG), and macrophage colony-stimulating factor (M-CSF), indicating that the LPS-TLR4-dependent survival of osteoclasts due to LPS-TLR4 is mediated by the TRAF6-NF-κB and PI3K-AKT pathways.

The role of internal TLRs in host cells is currently understood to be the induction of inflammation. Several DAMPs derived from dead cells have been reported to function as endogenous TLR4 ligands, including high-mobility group box 1 (HMGB1) and S100 proteins, which induce osteoclastic bone resorption [42,43,44]. HMGB1 can also bind to TLR2 and TLR5, which activate inflammatory responses [45]. These in vivo and in vitro studies demonstrate that TLR4 signaling driven by LPS and endogenous factors is a potent inducer of bone resorption through an indirect effect mediated by RANKL expression in osteoblasts and a direct effect that extends the lifespan of osteoclasts (Figure 2).

### 3.3. Cell Surface TLR2 Heterodimers as Receptors for Bacterial Cell-Wall Components from Gram-Positive and Gram-Negative Bacteria

TLR2 forms heterodimers with TLR1 or TLR6 to recognize lipopeptides and lipoteichoic acids (LTAs) of Gram-positive or Gram-negative bacteria membrane components and activates MyD88-mediated signaling [46].

Gram-positive bacteria are found in supragingival plaques, in contrast to Gram-negative bacteria. TLR2/1 heterodimers recognize bacterial triacylated lipopeptides, whereas the TLR2/6 heterodimer recognizes bacterial diacylated lipopeptides, such as LTA, macrophage-activating lipopeptide 2 (MALP2) from Mycoplasma fermentans, FSL-1 from M. salivarium, and zymosan, a β-glucan-rich product found in the yeast cell wall [46]. We previously reported that synthetic ligands for TLR2/1 (Pam3CSK4) and TLR2/6 (Pam2CSK4) induce osteoclast differentiation through PGE2 production in osteoblasts [20]. LTA derived from the cell wall of Gram-positive bacteria, a natural ligand for TLR2/6, induces PGE2-mediated osteoclast differentiation, whereas indomethacin treatment suppresses these activities [21], and TLR2 ligands extend the lifespan of mature osteoclasts [20,21]. Local injection of these ligands for TLR2 into the mandibular gingiva induces alveolar bone resorption in mice [20,21]. Based on these data, we suggest that TLR2 signaling, similar to TLR4 signaling in osteoblasts, is involved in PGE2-mediated osteoclast differentiation (Figure 2). Yano et al. reported that the TLR2/1 ligand (Pam3CSK4) directly induces osteoclast differentiation in Raw264.7 cells [47]. A recent report indicated that sialylated TLR2 functions as a receptor for sialic acid-binding immunoglobulin-type lectin 15 (Siglec15), which is identified as a ligand for DNAX-activating protein 12 kDa (DAP12) and initiates cell-to-cell fusion between preosteoclasts for osteoclast differentiation [48]. Thus, TLR2 signaling positively regulates the osteoclast differentiation and function.

### 3.4. Cell Surface TLR5, a Receptor for Flagellum from Gram-Positive and G-Negative Bacteria

Flagellin, a component of the flagellum of Gram-positive and Gram-negative bacteria, is recognized by TLR5. Ha et al. reported that TLR5 activation by flagellin suppresses RANKL-induced osteoclast differentiation by inhibiting c-Fos expression, a key regulator of osteoclast differentiation, and promoting interferon-β (IFN-β) production in bone marrow-derived macrophage cultures, whereas flagellin induces osteoclast differentiation in co-cultures of osteoblasts and bone marrow cells without inducing IFN-β production [49]. Kassem et al. reported that TLR5 activation by a local injection of flagellin over the skull bone in mice induces osteoclast differentiation and bone resorption associated with increased *Tnfsf11* (RANKL) mRNA expression [50]. Chamberlain et al. reported that potential endogenous TLR5 ligands, such as lectin and HSPs, promote TNF-α production from RA fibroblasts and macrophages to exacerbate RA progression [51]. Kim et al. reported that intra-articular injection of flagellin promoted TNF-α production through monocyte infiltration and exacerbated arthritic bone erosion in collagen-induced arthritis model mice [52]. These reports suggest that TLR5 signaling may be involved in periodontal alveolar bone resorption.

### 3.5. Intracellular TLR3, 7, and 9 as Receptors for Nucleic Acids Including Endogenous and Exogenous RNA and DNA

There are few reports on the roles of TLR3, 7, and 9 expressed on endosomal/lysosomal membranes in bone resorption. Mouse TLR8 has been suggested to be non-functional or to partially control TLR7 expression [53]. Kim et al. reported that poly(I:C) upregulates RANKL expression in RA fibroblast-like synoviocytes (FLS), and co-culture of human monocytes with TLR3-activated RA-FLS induces osteoclast differentiation [54]. We previously demonstrated that poly(I:C), a synthetic dsRNA analog, is endocytosed by osteoblasts and upregulates RANKL expression, inducing osteoclast differentiation [22]. Indomethacin and an EP4 antagonist blocked this effect, suggesting that PGE2 mediates poly(I:C)-TLR3 signaling-induced osteoclast differentiation (Figure 2) [22]. TLR3 also recognizes RNA from dead cells, mitochondrial dsRNA, and viral dsRNA [55,56,57]. Because these RNAs are derived from host dead cells, including necrotic cells, apoptotic cells, and immune cells, releasing antimicrobial extracellular traps, and infected bacteria may accumulate in inflamed tissues, TLR3 signaling may be involved in inflammatory bone resorption in periodontitis and others.

The synthetic TLR7 ligand, B848 (resiquimod) or bropirimine, has been reported to inhibit RANKL-induced osteoclast differentiation, but not the survival or function, in BMM cultures and human PBMC cultures [58] and suppresses di-hydroxyvitamin D3 [1,25(OH)2D3]-induced osteoclast differentiation in co-cultures of bone marrow cells and osteoblasts through IFN-β production [59]. Several reports have shown that TLR7 signaling contributes to inflammatory bone resorption associated with RA. Alzabin et al. reported that TLR7-deficient mice showed milder pathology of collagen-induced arthritis than wild-type mice [60]. TLR7 activated by single-stranded RNA from RA synovial fluid has been reported to promote osteoclast differentiation by upregulating RANKL expression in synovial fibroblasts [61]. Hegewald et al. reported that miR-574-5p delivered by small extracellular vesicles secreted from synovial fibroblasts in RA patients induces osteoclast differentiation via TLR7/8 signaling [62].

Synthetic oligonucleotides containing unmethylated CpG dinucleotides (CpG-ODNs) promote osteoclast differentiation from RANKL-pretreated BMMs via the induction of TNF-α, whereas CpG-ODN inhibits osteoclast differentiation from BMMs via the downregulation of M-CSF receptor at the early stage of differentiation [63,64]. Ding et al. reported that osteoclast differentiation is promoted in cell cultures of TLR9-deficient BMMs treated with M-CSF and RANKL, and TLR9-deficient mice exhibit low bone mass due to the promotion of osteoclast differentiation, which is associated with chronic systemic inflammation [65]. Kim et al. demonstrated that TLR9 signaling elevates proinflammatory cytokine production, including IL-6 and TNF-α, and mediates inflammatory alveolar bone loss induced by *P. gingivalis* infection in mice [66].

These reports suggest that intracellular TLRs may be involved in the pathogenesis or progression of periodontal bone resorption by recognizing bacterial or host cell-derived nucleic acids.

### 3.6. TLR Ligands Bind TLRs and Coordinate the Inflammatory Signaling with Other Family of Cytosolic Sensor

Several TLR ligands are incorporated into the cytosol of host cells through endocytosis of bacterial extracellular vesicles or a type III secretory system by certain bacteria and are recognized by cytosolic PRRs, which activate TLR-independent signaling. LPS is one of the components of bacterial outer membrane vesicles (OMVs) secreted from Gram-negative bacteria, and OMVs are endocytosed by cells, delivering LPS into the cytosol [67,68]. Intracellular LPS directly binds to caspase-11, which cooperates with NOD-like receptor protein 3 (NLRP3) to trigger caspase-11-dependent cell death and IL-1β responses. Flagellin is also delivered to the cytosol via the type III secretory system [69]. Intracellular flagellin is sensed by the NOD-like receptor family caspase recruitment domain-containing protein 4 (NLRC4), which activates the NLRC4 inflammasome and caspase-1-mediated IL-1β secretion [70]. Recent studies have shown that NLRP3 and NLRC4 inflammasomes positively regulate osteoclast differentiation and bone resorption [71,72].

In contrast, RNA and DNA are sensed by cytosolic sensors after endosomal escape. Retinoic acid-inducible gene-I (RIG-I) and melanoma differentiation-associated protein 5 (MDA5) preferentially recognize short (<300 bp) and long (>1000 bp) dsRNAs, respectively, leading to mitochondrial antiviral signaling (MAVS)-dependent NF-κB and IRF activation [73]. The cyclic GMP-AMP synthase (cGAS)-stimulator of interferon genes (STING) pathway senses cytosolic DNA, including mitochondrial DNA, as well as bacteria and dead cells, which activate the transcription of NF-κB and IRF [74]. MacLauchlan et al. reported that STING-deficient mice show bone loss over time due to IFN-dependent suppression of osteoclast differentiation [75].

Further studies are required to clarify the crosstalk between TLRs and cytosolic sensors activated by TLR ligands that regulate bone resorption.

### 3.7. Roles of Immune Cells Activated by TLR Ligands in Inflammatory Bone Resorption

Various immune cells are infiltrated and accumulated in infected periodontal tissues to regulate local immune responses. Li et al. profiled and analyzed the subtypes of immune cells present in periodontitis tissues (n = 210) compared to healthy periodontal tissues (n = 133) using datasets from the Gene Expression Omnibus (GEO) [76]. They showed that plasma cells (differentiated B cells), naïve B cells, and neutrophils are elevated in periodontitis tissues, whereas memory B cells, CD4^+^ memory T cells, dendritic cells, mast cells and M1/M2 macrophages are more prevalent in healthy tissues. These immune cells can contribute to osteoclast differentiation by producing inflammatory cytokines and RANKL in inflammatory conditions [77,78,79,80]. Several reports demonstrated that neutrophils, B cells and T cells accumulated in periodontal lesions produce inflammatory mediators [81,82], including TNF-α, IL-1, PGE2 and oncostatin. M. Makkawi et al. demonstrated that TLR2/PI3K signaling activated by *P. gingivalis* in neutrophils and macrophages facilitates the production of inflammatory cytokines [83]. Inflammatory mediators support osteoclast differentiation and alveolar bone resorption through upregulating RANKL expression in osteogenic cells, such as osteoblasts and periodontal ligament cells, and/or directly activating osteoclasts. Chakravarti et al. reported that several TLR ligands for TLR4 (LPS), TLR2 (Pam3CSK4), TLR5 (flagellin) and TLR7 (gardiquimod) induce RANKL expression in neutrophils, and osteoclast differentiation is induced in co-cultures of monocytes and LPS-activated neutrophils without the addition of exogenous RANKL [77]. Also, neutrophil extracellular traps (NETs), which are net-like structures composed of DNA and antimicrobial proteins to trap and kill pathogens, have been reported to exacerbate alveolar bone resorption in periodontal disease [84,85]. TLR ligands for TLR2/6, TLR4, TLR7, TLR8 and TLR9 induce NET formation through NADPH oxidase (NOX)-dependent ROS production [86]. On the other hand, some immune cells, including regulatory B cells (Bregs), regulatory T cells and CD8^+^ T cells, have been reported to inhibit inflammatory alveolar bone resorption [87,88,89].

Overall, in immune-bone interaction, certain immune cells promote bone resorption and others inhibit it, indicating that a disrupted balance of immune cell populations in periodontitis tissues contributes to the development of periodontal bone resorption.

## 4. Conclusions

**Figure 3:**
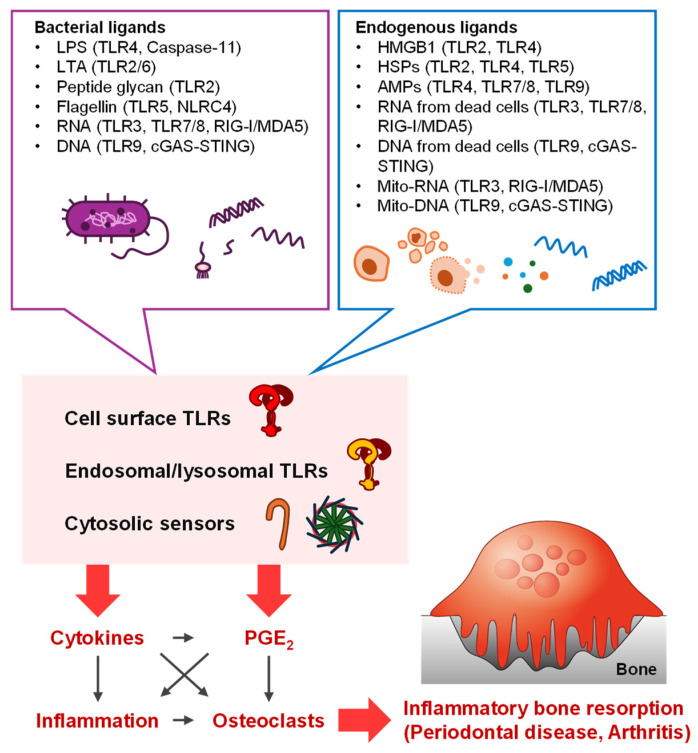
Schematic illustration of inflammatory bone resorption induced by TLR ligands. In inflammatory tissues, such as periodontal tissues infected with bacteria and arthritic joints, bacterial ligands derived from infected bacteria and endogenous ligands derived from host cells, such as immune cells and stromal cells, are recognized by the cell surface and endosomal/lysosomal TLRs and cytosolic sensors (RIG-I/MDA5, cGAS-STING, and inflammasomes), stimulating the production of inflammatory cytokines and PGE2. These factors induce osteoclast differentiation and bone resorption, which are associated with inflammation.

Current scientific progress in the field of infectious inflammation and TLRs has revealed the inevitable roles of TLR signaling in inflammatory bone resorption of periodontal disease. LPS derived from Gram-negative bacteria is a major pathogen of periodontal alveolar bone resorption via TLR4 signaling, and other TLRs contribute to the elongation of TLR4 functions. TLR ligands from bacterial membranes, including LPS and LTA (TLR2/1, TLR2/6, and TLR4 ligands), flagellin (TLR5 ligand), and nucleic acids (TLR3, TLR,7, and TLR9 ligands), are cross-sectionally recognized by all TLRs. Endogenous factors, such as DAMPs from dead cells and components of extracellular traps from immune cells recruited to inflamed tissues, are also recognized by TLRs. These TLR ligands are also sensed by cytosolic sensors, such as inflammasomes, RIG-I/MDA5, and cGAS-STING. The signaling networks in the pathogenic bone and surrounding tissues are presumed to be highly active and increase inflammation and subsequent inflammatory bone resorption (Figure 3).

The role of TLR signaling in osteoclasts remains controversial. Osteoclast precursors express TLR1-9, especially TLR2 and TLR4, which are the major TLRs induced by RANKL in mature osteoclasts [21,22,26]. We previously screened which TLR ligands induced osteoclast differentiation in co-cultures of mouse primary osteoblasts (POBs) and bone marrow cells (BMCs) among TLR2/1 (Pam3CSK4), TLR2/6 (fibroblast-stimulating lipopeptide 1; FSL-1), TLR3 (poly[I:C]), TLR4 (LPS from *Escherichia coli* and *Porphyromonas gingivalis*), TLR5 (flagellin from *Salmonella typhimurium*), TLR7 (single-strand RNA 40), and TLR9 (CpG oligonucleotide; ODN1826). The ligands for TLR2/1, TLR2/6, TLR3, TLR4, and TLR5 significantly induced osteoclast differentiation (Figure 2). The inhibitory effects of TLR ligands have been reported for TLR2 (peptidoglycan from *Staphylococcus aureus*), TLR3 (poly[I:C]), TLR4 (LPS from *E. coli*), and TLR9 (synthetic phosphothioate-stabilized CpG DNA), which strongly inhibited RANKL-induced osteoclast differentiation despite stimulation of tumor necrosis factor-α (TNF-α) in mouse bone marrow macrophage (BMM) cultures and the mouse macrophage lineage cell line Raw264.7 [26]. In contrast, TLR2 and TLR4 activation by each ligand has been shown to enhance the survival of mature osteoclasts in BMM cultures [26]. These reports suggest that several TLRs play different roles in inflammatory bone resorption, including in periodontitis. Further investigation is needed to elucidate the precise role of TLRs in osteoclast differentiation.

## 5. Future Perspectives

Bacterial infection activates TLR signaling and other cytosolic sensor signaling through bacterial components and autologous DAMPs released from host-derived immune cells and dead cells in the periodontal tissues. These factors participate in the development and progression of inflammatory bone resorption (Figure 3). Further research is needed to elucidate the interaction networks of each TLR, including both endosomal/lysosomal TLRs and other cytosolic sensor families, activated by exogenous and endogenous ligands originating from bacteria and host cells. These findings concerning TLR function are expected to contribute to effective drug discovery for progressive periodontal diseases.

## Figures and Tables

**Table 1:** TLR ligands.

TLR	Location	Exogenous Ligands (MAMPs)	Endogenous Ligands (DAMPs)
TLR2/1 and/or TLR2/6	Cell surface (Cell membrane)	Peptide glycan	Amyloid, HSPs, HMGB1, Hyaluronan
TLR2/1	Cell surface (Cell membrane)	Triacylated lipopeptide	-
TLR2/6	Cell surface (Cell membrane)	Diacylated lipopeptide, LTA, Fungal zymosan	-
TLR3	Endosome/lysosome	dsRNA	snRNA, Mitochondrial dsRNA, RNA from dead cells
TLR4	Cell surface (Cell membrane)	LPS, Virus envelope protein	Oxidized LDL, AGE-LDL, HSPs, HMGB1, Hyaluronan, S100 proteins, Fibrinogen, Anti-MPs
TLR5	Cell surface (Cell membrane)	Flagellin Fungal zymosan	Lectin, HSPs
TLR7	Endosome/lysosome	ssRNA	RNA from dead cells Anti-MPs
TLR8	Endosome/lysosome	ssRNA	RNA from dead cells Anti-MPs
TLR9	Endosome/lysosome	Unmethylated CpG DNA	HMGB1, Mitochondria DNA, Anti-MPs
TLR10	Endosome/lysosome	-	-
TLR11	Cell surface (Cell membrane)	Profilin-like protein	-
TLR12	Endosome/lysosome	Profilin	-
TLR13	Endosome/lysosome	23S rRNA	-

HSP: Heat-shock protein, HMGB1: high-mobility group box 1, LTA: lipoteichoic acid, dsRNA: double-stranded RNA, snRNA: small nuclear RNA, LPS: lipopolysaccharide, LDL: low-density lipoprotein, AGE: advanced glycation end product, Anti-MP: antimicrobial peptide, ssRNA: single-strand RNA.

## Data Availability

The raw data supporting the conclusions of this article will be made available by the authors on request.

## Abbreviations

**Abbreviation or Acronym**	**Name**
1,25(OH)2D3	1,25-dihydroxyvitamin D3
AA	Arachidonic acid
AGE	Advanced glycation end product
*A. Actinomycetemcomitans*	*Aggregatibacter actinomycetemcomitans*
Anti-MP	Antimicrobial peptide
AP-1	Activator protein-1
BMC	Bone marrow cell
BMD	Bone mineral density
CD	Cluster of differentiation
cGAS	Cyclic GMP-AMP synthase
COX	Cyclooxygenase
ODN	Oligodeoxynucleotide
DAMP	Damage-associated molecular pattern
DAP12	DNAX activating protein 12 kDa
dsRNA	Double strand RNA
*E. coli*	*Escherichia coli*
EP	Prostaglandin E2 receptor
FLS	Fibroblast-like synoviocyte
FSL-1	Fibroblast-stimulating lipopeptide
GEO	Gene Expression Omnibus
HMGB1	High mobility group box 1
HSP	Heat shock protein
IFN	Interferon
IκBα	Inhibitor of NF-κB α
IKK	IκB kinase
IL	Interleukin
IRAK	IL-1 receptor-associated kinase
IRF	Interferon regulatory factor
LDL	Low-density lipoprotein
LL37	Cathelicidin-related antimicrobial peptide
LPS	Lipopolysaccharide
LTA	Lipoteichoic acid
MAMP	Microbe-associated molecular pattern
MALP2	Macrophage-activating lipopeptide 2
MAPK	Mitogen-activated protein kinase
M-CSF	Macrophage colony-stimulating factor
MD2	Myeloid differentiation protein 2
MDA5	Melanoma differentiation-associated gene 5
mPGES-1	Membrane-bound PGE synthase
MyD88	Myeloid differentiation factor 88
NFATc1	Nuclear factor of activated T cells, cytosolic 1
NET	Neutrophil extracellular trap
NF-κB	Nuclear factor-κB
NLRC	NOD-like receptor family caspase recruitment domain-containing protein 4
NLRP	NOD-like receptor protein 3
NOD	Nucleotide oligomerization domain
NOX	NADPH oxidase
OMV	Bacterial outer membrane vesicle
OPG	Osteoprotegerin
PAMP	Pathogen-associated molecular pattern
PG	Prostaglandin
PI3K	Phosphatidylinositol-3 kinase
PLA2	Phospholipase A2
*P. gingivalis*	*Porphyromonas gingivalis*
PRR	Pattern recognition receptor
RA	Rheumatoid arthritis
RANK	Receptor activator of NF-κB
RANKL	Receptor activator of NF-κB ligand
RIG-I	Retinoic acid-inducible gene-I
RIP	Receptor interacting protein
snRNA	Small nuclear RNA
ssRNA	Single-strand RNA
STING	Stimulator of interferon genes
TIRAP	Toll/interleukin-1 receptor-domain-containing adapter protein
TLR	Toll-like receptor
TNF	Tumor necrosis factor
TRAF	TNF receptor-associated factor
TRAM	TRIF-related adaptor molecule
TRIF	Toll/interleukin-1 receptor-domain-containing adapter-inducing interferon-β
